# Incidence and predictors of clinical failure after early treatment for mild‐to‐moderate COVID‐19 in high‐risk individuals: A multicentric cohort study

**DOI:** 10.1111/joim.20030

**Published:** 2024-11-13

**Authors:** Ilaria Mastrorosa, Alessandro Cozzi Lepri, Cosmo Del Borgo, Silvia Rosati, Martina Rueca, Loredana Sarmati, Claudio Mastroianni, Massimo Fantoni, Fabrizio Maggi, Emanuele Nicastri, Enrico Girardi, Miriam Lichtner, Andrea Antinori, Valentina Mazzotta, the Early Treatment for COVID‐19 Lazio Study Group

**Affiliations:** ^1^ Clinical and Research Infectious Diseases Department National Institute for Infectious Diseases Lazzaro Spallanzani IRCCS Rome Italy; ^2^ Centre for Clinical Research Epidemiology Modelling and Evaluation (CREME), Institute for Global Health, UCL London UK; ^3^ Infectious Diseases Unit Santa Maria Goretti Hospital Sapienza University of Rome Latina Italy; ^4^ Laboratory of Virology National Institute for Infectious Diseases Lazzaro Spallanzani IRCCS Rome Italy; ^5^ Infectious Diseases Unit Tor Vergata University Hospital Rome Italy; ^6^ Department of Public Health and Infectious Diseases Sapienza University of Rome Rome Italy; ^7^ Emergency Infectious Diseases Unit Fondazione Policlinico Universitario A. Gemelli IRCCS Rome Italy; ^8^ Scientific Direction National Institute for Infectious Diseases Lazzaro Spallanzani IRCCS Rome Italy; ^9^ PhD Course in Microbiology, Immunology, Infectious Diseases, and Transplants (MIMIT) Infectious Diseases University of Rome Tor Vergata Rome Italy

**Keywords:** COVID‐19 early treatment, COVID‐19‐related death, COVID‐19‐related hospitalization, immunocompromised status, older age

## Abstract

**Objectives:**

To estimate the risk of COVID‐19‐related hospitalization and death (CovH/D), among high‐risk individuals early treated for COVID‐19 and to identify associated factors.

**Methods and results:**

A multicenter cohort of 12,475 high‐risk outpatients (female 50.2%, median age 70 years [IQR 57–80], fully vaccinated 79.1%, immunocompromised 23.2%) treated with monoclonal antibodies or antivirals for mild‐to‐moderate COVID‐19 (March 2021–May 2023) in the Lazio region, Italy. The unadjusted risk of CovH/D by Day 30 was 3.08% (95% CI 2.7%–3.4%). By means of logistic regression models, which included a specific set of potential confounders for each exposure of interest, we observed a higher risk for the elderly, unvaccinated and immunocompromised participants. Using the “Delta period” as a reference, a decreased risk was observed for Omicron waves.

**Conclusions:**

Despite the administration of COVID‐19 early treatment and the decreasing risk of CovH/D across the calendar periods, the elderly, the unvaccinated and the immunocompromised people remain at high risk of clinical progression.

## Introduction

At the end of 2019, a novel coronavirus designated severe acute respiratory syndrome coronavirus 2 (SARS‐CoV‐2), rapidly spread throughout the world, resulting in a global pandemic with over 750 million confirmed cases of coronavirus disease 2019 (COVID‐19) [1]. The spectrum of COVID‐19 disease in adults ranges from asymptomatic infection to mild respiratory tract symptoms to severe pneumonia. The risk of severe illness varies by age, underlying comorbidities, immunity due to prior SARS‐CoV‐2 infection and vaccination *status* [2]. Additionally, viral variants have been associated with heterogeneous risks of severe disease, estimated as lower with Omicron [3]. The management of COVID‐19 in the outpatient setting during the early phases of the acute infection and the correct identification of individuals at increased risk for hospitalization or death are crucial to prevent disease progression. Early treatment with antivirals or monoclonal antibodies (mAbs) remains a high priority in high‐risk, not‐hospitalized patients, as reported by national and international recommendations [4, 5], albeit with some differences according to specific country [6]. Although, at the present state of the pandemic, the spread of vaccination and the lower pathogenicity of the current SARS‐CoV‐2 variants have drastically reduced the rate of COVID‐19‐related complications, there are restricted categories of individuals who remain at risk of disease progression, even after receiving prompt treatment. There is a lack of real‐world data aimed to identify these specific subgroups, which can help detect people at greater risk of progression to severe disease and death and inform guidelines for COVID‐19 early treatment strategies and vaccination programs in fragile populations in the Omicron era.

In our real‐life cohort of high‐risk individuals early treated with antivirals or mAbs over a period covering a range of SARS‐CoV‐2 variants of concern (VoCs), we aimed to estimate the risk of hospitalization and death in this target population and identify factors associated with the risk of progression to severe COVID‐19 disease.

## Methods

### Study design and study procedures

The present report uses the data of a prospective multicentre observational study (including five infectious disease centers in the Lazio region of Italy) on the effectiveness of early treatment with mAbs or antiviral agents for high‐risk outpatients with mild‐to‐moderate COVID‐19. Herein, we included all the patients consecutively enrolled, from March 2021 to May 2023, in the overmentioned five hospitals and treated with antivirals (molnupiravir, remdesivir and nirmatrelvir/ritonavir) or mAbs (bamlanivimab, bamlanivimab/etesevimab, casirivimab/imdevimab, sotrovimab and tixagevimab/cilgavimab) according to AIFA eligibility criteria [5], the physician's assessment and judgment, the availability of drugs (which has changed over time) and the presence of contraindications to specific drugs.

On the day of the first evaluation, demographic and clinical data were collected, including information on time from symptoms onset, vaccination status and comorbidities, and treatment was administered. All the data were anonymously collected into an electronic database. The SARS‐CoV‐2 variant was established either directly, using base sequencing where available, or indirectly, inferred from the calendar period of baseline date based on Italian regional surveillance data. Data on vaccination were extracted from the regional register (Anagrafe Vaccinale Regione Lazio) and, if not available, we collected self‐reported vaccination status from clinical charts. Participants were defined with (1) none or incomplete vaccination, if they did not receive any vaccine dose, or they received less than three doses; (2) full but not recent vaccination, if they received more than three doses with the last dose administered more than 120 days before; (3) full recent vaccination, if they received more than three doses with the last dose administered less than 120 days before. Participants were followed up to 30 days by in‐presence or telephonic visits, and information on hospitalization or death was collected.

### Statistical analysis

The primary endpoint was the proportion of participants who experienced COVID‐19‐related clinical failure, defined as hospitalization due to the development of severe COVID‐19 or death from any cause over Days 0–30.

The main characteristics of the study population assessed at baseline were described using medians and interquartile ranges (IQR) for continuous variables and frequencies and percentages for categorical variables. The unadjusted risk of clinical failure by Day 30 was calculated with 95% CI. We also fitted a logistic regression analysis with the binary endpoint of hospitalization and death and four primary exposures of interest measured at baseline: (1) older age (≥75 years old), (2) vaccination status (a. full recent primary cycle and booster dose, b. full not recent primary cycle and booster dose, c. less than three doses of vaccination), (3) immunocompromised status (defined as primary or secondary immunodeficiency and/or active treatment with immunosuppressive agents) and (4) calendar time of entry in care stratified according to the periods in which the various VoCs were circulating. A separate model for these exposures, including a specific set of potential confounders, was fitted. We also hypothesized at the outset that the effect of age and immunodeficiency may vary by pandemic wave (pre‐ vs. post‐Omicron circulation). Therefore, we have formally tested these interactions and reported stratified analyses if there was evidence for effect measure modification. All analyses have been performed using SAS version 9.4.

## Results

A total of 12,475 individuals were enrolled. Table 1 shows the study population's main characteristics. Briefly, 6259 (50.2%) were females, the median age was 70 years (IQR 57–80), and 4714 (37.8%) subjects were 75 or older. Participants were mainly fully vaccinated (9858, 79.1%), whereas 2608 (20.9%) were those with none or incomplete vaccination. Subjects with cardio‐metabolic comorbidity (such as obesity, diabetes and cardiovascular disease) were 7885 (63.3%), and those with an immunocompromised *status* were 2892 (23.2%).

**Table 1:** Characteristics of the study population.

*Characteristics*	Total (*n* = 12,475)
***Gender, n (%)***	
Female	6263 (50.2%)
***Age, years***	
Median (IQR)	70 (57, 80)
***Vaccination, n (%)***	
None or incomplete	2618 (21.0%)
Full, not recent (last dose older than 120 days ago)	6598 (52.9%)
Full, recent (last dose less than 120 days ago)	3259 (26.1%)
***Comorbidities, n (%)***	
Cardio‐matabolic (obesity, diabetes, CVD)	7889 (63.2%)
Hepatic disease	264 (2.1%)
Cancer	1001 (8.0%)
Immunocompromised *status* ^a^	2898 (23.2%)
Diabetes alone	2116 (17.0%)
Neurologic	241 (1.9%)
Renal	1299 (10.4%)
COPD	2582 (20.7%)
***VoC, n (%)***	
Alpha	381 (3.1%)
Delta	1722 (13.8%)
BA.1	3133 (25.1%)
BA.2	2963 (23.8%)
BA.4/5	3681 (29.5%)
BQ.1	393 (3.2%)
XBB.1.5	202 (1.6%)
***Viral sequence available, n (%)***	
No	10,209 (81.8%)
***Treatment, n (%)***	
BAM	11 (0.1%)
BAM/ETE	1304 (10.5%)
CAS/IMD	1009 (8.1%)
TIX/CIL	433 (3.5%)
MLP	3734 (29.9%)
NMV/r	2509 (20.1%)
RDV	1447 (11.6%)
SOT	2028 (16.3%)

Abbreviations: BAM, bamlanivimab; BAM/ETE, bamlanivimab/etesevimab; CAS/IMD, casirivimab/imdevimab; CVD, cardiovascular diseases; IQR, interquartile range; MLP, molnupiravir; n, number of participants; NMV/r, nirmatrelvir/ritonavir; RDV, remdesivir; SOT, sotrovimab; TIX/CIL, tixagevimab/cilgavimab; VoC, variant of concern.

^a^ Primary or secondary immunodeficiency and/or active treatment with immunosuppressive agents.

A total of 384 events (COVID‐19‐related hospitalization/death) were observed by Day 30 from treatment initiation in our sample, with an estimated incidence of 3.08% (95% CI 2.7%–3.4%).

After controlling for potential confounders, a higher risk was observed for age (odds ratio [OR] 2.33 per 10 years older; 95% CI 1.87–2.90), unvaccinated (OR 2.03 vs. fully vaccinated; 95% CI 1.51–2.73) and immunocompromised (OR 1.36 vs. immunocompetent; 95% CI 1.03–1.78) (Table 2). The association with immunosuppression was even stronger when restricting the analysis to the “Omicron period” (OR 1.72, 95% CI 1.28–2.30, interaction *p* value = 0.01) (Table 3). In contrast, there was no evidence for effect measure modification by age (*p* = 0.22). Using the “Delta period” as the comparator, a decreased risk was observed across all more recent periods when Omicron sublineages were circulating (Table 2).

**Table 2:** Factors associated with COVID‐19‐related clinical failure, defined as hospitalization due to severe COVID‐19 or death from any cause.

	Hospitalized/dead *n* (%)	Recovered n (%)	Unadjusted OR (95% CI)	*p* value	Adjusted1^a^ OR (95% CI)	*p* value	Adjusted2^b^ OR (95% CI)	*p* value
***Age***								
18–74	142 (41.5%)	7327 (62.3%)	1					
≥75	200 (58.5%)	4425 (37.7%)	2.33 (1.87, 2.90)	<0.001				
***Vaccination***								
Full, recent^c^	81 (23.7%)	3162 (26.9%)	1		1		1	
Full, not recent^d^	153 (44.7%)	6445 (54.8%)	0.93 (0.71, 1.22)	0.584	0.93 (0.71, 1.22)	0.589	0.94 (0.71, 1.23)	0.635
None or incomplete^e^	108 (31.6%)	2145 (18.3%)	1.97 (1.47, 2.64)	<0.001	2.09 (1.56, 2.81)	<0.001	2.03 (1.51, 2.73)	<0.001
***VoC***								
Delta	87 (25.4%)	1635 (13.9%)	1		1		1	
BA.1	102 (29.8%)	3031 (25.8%)	0.63 (0.47, 0.85)	0.002	0.54 (0.40, 0.72)	<0.001	0.73 (0.54, 0.98)	0.036
BA.2	53 (15.5%)	2910 (24.8%)	0.34 (0.24, 0.48)	<0.001	0.27 (0.19, 0.39)	<0.001	0.36 (0.26, 0.51)	<0.001
BA.4/5	85 (24.9%)	3596 (30.6%)	0.44 (0.33, 0.60)	<0.001	0.35 (0.25, 0.47)	<0.001	0.46 (0.34, 0.62)	<0.001
BQ.1	9 (2.6%)	384 (3.3%)	0.44 (0.22, 0.88)	0.021	0.32 (0.16, 0.65)	0.002	0.47 (0.23, 0.94)	0.032
XBB.1.5	6 (1.8%)	196 (1.7%)	0.58 (0.25, 1.33)	0.197	0.49 (0.21, 1.13)	0.093	0.60 (0.26, 1.38)	0.226
***Immunocompromised status*** ^f^								
No	266 (77.8%)	8986 (76.5%)	1		1		1	
Yes	76 (22.2%)	2766 (23.5%)	0.93 (0.72, 1.20)	0.572	1.23 (0.94, 1.61)	0.133	1.36 (1.03, 1.78)	0.027

Abbreviations: CI, confidence interval; *n*, number; OR, odds ratio; VoC, variant of concern.

^a^ Adjusted for gender and age.

^b^ Separate adjustments for each exposure: – vaccination—for age, immunocompromised *status* and calendar year, – VOC—for vaccination, – immunocompromised *status*—for age, vaccination and calendar year.

^c^ Last dose less than 120 days ago.

^d^ Last dose older than 120 days ago

^e^ Less than three doses.

^f^ Primary or secondary immunodeficiency and/or active treatment with immunosuppressive agents.

**Table 3:** Effect of immunodeficiency in different pandemic waves.

	Hospitalized/dead *n* (%)	Recovered *n* (%)	Unadjusted OR (95% CI)	Adjusted1^a^ OR (95% CI)	Adjusted2^b^ OR (95% CI)	Interaction *p* value 0.01
**Alpha/delta wave**						
***Immunocompromised status*** ^c^						
No	80 (92.0%)	1332 (81.5%)	1	1	1	
Yes	7 (8.0%)	303 (18.5%)	0.38 (0.18, 0.84)	0.45 (0.21, 1.00)	0.47 (0.21, 1.04)	
**Omicron wave**						
***Immunocompromised status*** ^c^						
No	186 (72.9%)	7654 (75.7%)	1	1	1	
Yes	69 (27.1%)	2463 (24.3%)	1.15 (0.87, 1.53)	1.72 (1.28, 2.30)	1.72 (1.28, 2.30)	

Abbreviations: CI, confidence interval; *n*, number; OR, odds ratio; VoC, variant of concern.

^a^ Adjusted for gender and age.

^b^ Adjusted for age, vaccination and calendar year.

^c^ Primary or secondary immunodeficiency and/or active treatment with immunosuppressive agents.

## Discussion

In our real‐world multicenter study, including more than 12,000 subjects who accessed the participating facilities for early treatment of COVID‐19 over 2 years (March 2021–May 2023), we estimated an overall incident risk of clinical failure of 3.08% (95% CI 2.7%–3.4%). This estimate is close to (if anything, slightly lower) that observed in experimental arms of clinical trials investigating antivirals and mAbs [7, 8, 9] in the pre‐Omicron era. A similar low incidence of severe outcomes after the early treatment with mAbs and antivirals in high‐risk outpatients has been described in several observational cohort studies conducted in both pre‐Omicron [10] and Omicron [11, 12, 13] eras. Importantly, the large study period of our analysis, which lasted until May 2023, allowed us to include more recent omicron sublineages not covered in the cited studies. Interestingly, we confirmed the low risk of clinical failure although most of the study participants harbored omicron sublineages, whose mutations in the spike protein, not present in the ancestral strains and other SARS‐CoV‐2 variants, led to an evolving escape to in vitro neutralizing activity of mAbs; in contrast, the antivirals seemed to retain their efficacy albeit with some differences by type [14, 15, 16]. Despite the high proportion of individuals with immunosuppression included in our cohort, the low estimated failure rate observed is consistent with the high prevalence of SARS‐CoV‐2 fully vaccinated participants (nearly 80%) included in the cohort and the majority of participants infected with omicron sublineages, which are suggested to cause less severe disease. Indeed, our data also carried evidence for a lower risk of COVID‐19‐related hospitalization and death per more recent calendar periods (when Omicron variants were circulating), consistent with data from previous studies suggesting lower pathogenicity of this VoC and its sublineages compared to those circulating during earlier waves of the pandemic [3, 17].

Importantly, our analysis also shows that age and, after controlling for confounding factors, incomplete vaccination and immunocompromised *status* were all associated with a higher risk of developing severe COVID‐19/death. Existing literature strongly supports the effect of vaccination in decreasing the risk of developing severe COVID‐19 disease and the association between older age and higher risk of severe COVID‐19 and mortality [2, 18]. Interestingly, our results support that these factors still predict a progression to severe disease in the context of early treatment. Similarly, a large population study has demonstrated that people with underlying immunocompromising conditions or those taking immunosuppressive drugs are more likely to develop severe COVID‐19 and may have persistent clinical complications and prolonged viral shedding in the Omicron era [19]. Our data are consistent with a 36% higher risk of progression to severe disease when compared to immunocompetent individuals despite having received early treatment for COVID‐19. To our knowledge, this is the largest study ever conducted on the target population of outpatients at high risk of progression to severe COVID‐19 who received early treatment, across different pandemic periods, including Omicron waves. Few other studies [11, 20, 21] have evaluated the association between demographics and clinical characteristics of these individuals concerning their risk of clinical outcome. However, in smaller sample sizes and including less recent viral variants.

Our analysis aimed to estimate the total effect of key predictors of hospitalization or death, which is why the treatment received (e.g., antivirals or mAbs, which typically lie on the causal pathway between patients’ demographics and clinical features and the risk of severe outcomes) has only been described but not included in the regression analysis.

Several limitations of the present study need to be mentioned. First, we cannot rule out residual and unmeasured confounding bias because of the study's observational nature, especially for vaccination and immunosuppression. Indeed, a significant limitation is that, while being aware of the increasing number of reinfections globally, we had no information on natural immunization, a potential common cause of vaccination, and outcomes. Moreover, having collected self‐reported vaccination status when data were unavailable on the regional register could have introduced further bias (due to misclassification of exposure and residual confounding for the other associations of interest). In addition, despite data harmonization between the five centers, we must consider some degree of variability in how exposure has been precisely defined. Finally, although individuals at high risk of progression receiving early treatment is the ideal target population to address the risk of hospitalization and death associated with immunosuppression and other risk factors, we acknowledge that our results are not generalizable to different settings.

In conclusion, our findings show that, although we have observed in recent years an overall reduction in the risk of severe COVID‐19 disease, older age, lack of complete vaccination coverage, and established immunosuppression seem to remain important risk factors for COVID‐19 morbidity and mortality despite timely administration of early treatment for mild‐to‐moderate COVID‐19, even with most recent Omicron subvariants. Indeed, our data suggest that the immunocompromised *status* emerged as a critical risk factor for severe disease/death, especially in the Omicron wave of the pandemic. Since early treatment of COVID‐19, together with the implementation of SARS‐CoV‐2 vaccination campaigns, mainly in fragile populations, remains a cornerstone in the management of COVID‐19, our data are informative for targeting preventive strategies and tailoring therapies for patients carrying specific risk factors, in addition to emphasizing the need for improving access to early care.

## AUTHOR CONTRIBUTIONS

*Conceptualization*: Ilaria Mastrorosa, Andrea Antinori and Valentina Mazzotta. *Data curation and formal analysis*: Alessandro Cozzi Lepri. *Funding acquisition*: Enrico Girardi, Emanuele Nicastri and Andrea Antinori. *Investigation*: Martina Rueca and Fabrizio Maggi. *Methodology*: Ilaria Mastrorosa, Alessandro Cozzi Lepri Andrea Antinori and Valentina Mazzotta. *Project administration and supervision*: Andrea Antinori and Valentina Mazzotta. *Resources*: Ilaria Mastrorosa, Cosmo Del Borgo, Silvia Rosati, Enrico Girardi, Loredana Sarmati, Claudio Mastroianni, Massimo Fantoni, Emanuele Nicastri, Miriam Lichtner and Valentina Mazzotta. *Writing—original draft*: Ilaria Mastrorosa, Alessandro Cozzi Lepri, Andrea Antinori and Valentina Mazzotta. *Writing—review and editing*: Cosmo Del Borgo, Silvia Rosati, Martina Rueca, Enrico Girardi, Loredana Sarmati, Claudio Mastroianni, Massimo Fantoni, Fabrizio Maggi, Emanuele Nicastri and Miriam Lichtner. All authors agreed with and approved the final version of the manuscript.

## Conflict of interest statement

A.A. and V.M. have served as pay consultants to G.S.K., AstraZeneca, Pfizer and Gilead Sciences.

The other authors declare no conflicts of interest for the present study.

## Funding information

This study was supported by funds to the National Institute for Infectious Diseases “Lazzaro Spallanzani”, IRCCS, Rome (Italy), from Italian Ministry of Health (Programme CCM 2020; Ricerca Corrente—Linea 1 on emerging and re‐emerging infections). Alessandro Cozzi‐Lepri work is supported by EuCARE project funded by the EU under the HORIZON Europe programme, Grant agreement no. 101046016.

## Ethics statement

All included individuals have signed a written informed consent to participate in the study. The observational multicentric study protocol and the informed consent were approved by the Scientific Committee of the Italian Medicines Agency (AIFA) and by the Ethical Committee of the National Institute for Infectious Diseases “Lazzaro Spallanzani” in Rome, Italy, as National Review Board for COVID‐19 pandemic in Italy (approval number: n. *380, 30/09/2021. FAV del Registro delle Sperimentazioni 2020/2021*).

## Data Availability

Anonymized participant data will be made available upon reasonable requests directed to the corresponding author.
